# Research progress on non-protein-targeted drugs for cancer therapy

**DOI:** 10.1186/s13046-023-02635-y

**Published:** 2023-03-14

**Authors:** Yiwen Zhang, Lu Lu, Feifeng Song, Xiaozhou Zou, Yujia Liu, Xiaowei Zheng, Jinjun Qian, Chunyan Gu, Ping Huang, Ye Yang

**Affiliations:** 1 Center for Clinical Pharmacy, Cancer Center, Department of Pharmacy, Zhejiang Provincial People’s Hospital, Affiliated People’s Hospital, Hangzhou Medical College, 158 Shangtang Road, Hangzhou, 310014 Zhejiang China; 2 Key Laboratory of Endocrine Gland Diseases of Zhejiang Province, 158 Shangtang Road, Hangzhou, 310014 China; 3 School of Medicine & Holistic Integrative Medicine, Nanjing University of Chinese Medicine, 138 Xianlin Road, Nanjing, 210023 China

**Keywords:** Cancer therapy, Oligonucleotide drugs, mRNA vaccines, Target, Delivery

## Abstract

Non-protein target drugs, especially RNA-based gene therapies for treating hereditary diseases, have been recognized worldwide. As cancer is an insurmountable challenge, no miracle drug is currently available. With the advancements in the field of biopharmaceuticals, research on cancer therapy has gradually focused on non-protein target-targeted drugs, especially RNA therapeutics, including oligonucleotide drugs and mRNA vaccines. This review mainly summarizes the clinical research progress in RNA therapeutics and highlights that appropriate target selection and optimized delivery vehicles are key factors in increasing the effectiveness of cancer treatment in vivo.

## Introduction

Cancer treatment remains a challenge worldwide. Although overall survival is improved by surgical removal of tumor tissues, chemotherapy, and radiotherapy, recurrence and metastasis of cancers cannot be avoided [1]. Moreover, chemotherapy has serious adverse effects, such as systemic toxicity and multiple drug resistance, which require the development of novel and effective therapeutic drugs [2].

Small molecule agents and antibodies that can target intracellular or extracellular proteins in tumor cells have become increasingly popular because of their strong antitumor effects [3, 4]. However, they fail to block some transcription factors and oncoproteins, such as RAS [5], one of the most frequently mutated proteins in cancer. Therefore, non-protein-targeted drugs have emerged to address this dilemma. In particular, RNA-based drugs, which are important components of gene therapy, are the most notable and serve as potential therapeutics that can specifically target and silence any gene target [6]. The molecular weight of therapeutic RNAs is generally 7–20 kDa, which is much greater than that of small-molecule drugs (< 1 kDa) but less than that of antibodies (> 100 kDa). Full-length mRNA vaccines are also large (> 100 kDa) [7].

Owing to the development of and improvements in RNA technology, certain synthesized oligonucleotide drugs and macromolecular RNA drugs, such as antisense oligonucleotides (ASOs), small-interfering RNAs (siRNAs), and mRNA vaccines (Table 1), have been approved for marketing worldwide [8]. Additionally, an increasing number of oligonucleotide drugs (such as ASOs, siRNAs, and miRNAs) and mRNA drugs are entering clinical trials worldwide [9].

**Table 1:** Approved RNA-based drugs in market at present

Class	Drug name	Target	Disease	Year of approval
ASO	Nusinersen	Exon 7 of SMN2	SMA	2016
	Eteplirsen	Exon 51 of DMD	DMD	2016
	Inotersen	TTR mRNA	FAP	2018
	Volanesorsen	ApoC3	FCS	2019
	Golodirsen	Exon 53 of DMD	DMD	2019
	Vitolarsen	Exon 53 of DMD	DMD	2020
	Casimersen	Exon 45 of DMD	DMD	2021
siRNA	Patisiran	TTR mRNA	FAP	2018
	Givosiran	ALAS1 mRNA	AHP	2020
	Lumasiran	HAO1 mRNA	PH1	2020
	Inclisiran	PCSK9	Hypercholesterolaemia	2020
mRNA	BNT162b2	Spike protein	SARA-CoV-2	2020
	mRNA-1273	Spike protein	SARA-CoV-2	2020

*SMN2* survival pf motor neuron-2, *SMA* spinal muscular atrophy, *TTR* transthyretin, *FAP* familial amyloid polyneuropathy, *ApoC3* apolipoprotein C3, *FCS* Familial chylomicronemia syndrome, *DMD* duchene muscular dystrophy, *ALAS1* aminolevulinate synthase 1, *AHP* Acute hepatic porphyria, *HAO1* Hydroxyacid oxidase 1, *PH1* Primary hyperoxaluria type 1, *PCSK9* proprotein convertase subtilisin/kexin 9, *SARS-CoV-2* syndrome coronavirus 2

RNA-based drugs have played a important role in various diseases, ranging from genetic diseases to viral infections, and clinical studies on RNA-based therapeutics have yielded satisfactory results. Therefore, RNA molecules under development are potential candidates and powerful tools for cancer treatment [10]. This paper summarizes the research progress of the non-protein target drugs, mainly RNA-based drugs in cancer treatment in recent years, including oligonucleotide drugs (ASOs, siRNA, microRNA), and mRNA vaccines, and puts forward suggestions on the challenges brought by this class of new drugs, and fully exerts their therapeutic potential.

This paper summarizes the recent research progress on non-protein-targeted drugs, mainly RNA-based drugs, including oligonucleotide drugs (ASOs, siRNA, and microRNA) and mRNA vaccines, in cancer treatment and enumerates the current challenges faced by researchers studying this new class of drugs.

### Oligonucleotide therapeutics

Approximately 40 years ago, Paul Zamecnik and Mary Stephenson successfully used synthetic oligonucleotides to block the translation of viral RNA [11]. Currently, owing to the benefits of Watson–Crick base-pairing rules and maturation of RNA technologies, oligonucleotides can be used to treat diseases by binding to specific DNA or RNA sequences or proteins and interfering with target gene expression.

Oligonucleotide therapeutics are drugs consisting of 10–50 nucleotides in length, including ASOs, siRNAs, and microRNAs (miRNAs), and can regulate the post-transcriptional level and are expected to target special proteins that are otherwise difficult to target directly [12]. Hence, oligonucleotide therapeutics are considered the third pillar of drug development, after small-molecule drugs and antibodies [13]. Drug constructs based on the genomic sequences of target genes are simple to design, and drug candidates only require the identification of the target regions in the RNA associated with the disease process. The key is to design sequences that are highly specific to the target RNA and avoid hybridization with unexpected but homologous “bystander” RNAs. ASOs, siRNAs, and miRNAs are currently the most extensively studied drugs for treating malignant tumors. Here, we provide an overview of recent clinical research progress.

### Antisense oligonucleotide (ASO)

In 1978, Zamecnik and Stephenson used a 13-nucleotide ASO targeting the sequence of the Rous sarcoma virus to inhibit viral replication in vitro. This was the first study to report the therapeutic application of ASOs [11]. Subsequently, some commercial companies have focused on antisense therapeutics; thus, progress on oligonucleotide chemistry and formulations and the distribution and safety of ASOs have achieved satisfactory results (Table 2).

**Table 2:** Antisense oligonucleotides cancer therapeutics in clinal trials

Target	Drug name	Cancer	ClinicalTrials. gov Identifier	Current status
Bcl-2	Oblimersen	Solid tumors	NCT00543231	Phase I completed
	Oblimersen	Solid tumors	NCT00636545	Phase I completed
	Oblimersen plus carboplatin and paclitaxel	Advanced solid tumors	NCT00054548	Phase I completed
	Oblimersen plus etoposide and carboplatin	Lung cancer	NCT00017251	Phase I completed
	Olimersen plus paclitaxel	Lung cancer	NCT00005032	Phase I/II completed
	Olimersen plus Irinotecan	Colorectal cancer	NCT00004870	Phase I/II completed
	Oblimersen	CLL	NCT00021749	Phase I/II completed
	Oblimersen plus rituximab and fludarabine	CLL	NCT00078234	Phase I/II completed
	Oblimersen plus doxorubicin and docetaxel	Metastatic or locally advanced breast cancer	NCT00063934	phase I/II terminated
	Oblimersen plus docetaxel	Prostate cancer	NCT00085228	phase II completed
	Oblimersen with interferon alfa	mRCC	NCT00059813	Phase II completed
	Oblimersen plus dacarbazine	Melanoma	NCT00016263	Phase III completed
	Oblimersen plus dexamethasone	Multiple myeloma, plasma cell neoplasm	NCT00017602	Phase III completed
	Oblimersen plus fludarabine and cyclophosphamide	CLL	NCT00024440	Phase III completed
	BP1002	Advanced lymphoid malignancies	NCT04072458	Phase I recruiting
Grb2	BP1001 with or without LDAC	AML, CML, ALL, MDS	NCT01159028	Phase I completed
	BP1001-A plus paclitaxel	Advanced or recurrent solid tumors	NCT04196257	Phase I recruiting
	BP1001 plus ventoclax and decitabine	AML	NCT02781883	Phase II recruiting
CLU	OGX-011 with hormone therapy	Prostate cancer	NCT00054106	Phase I completed
	OGX-011 plus docetaxel	Metastatic or locally recurrent solid tumors	NCT00471432	Phase I completed
	OGX-011 plus docetaxel	Breast cancer	NCT00258375	Phase II completed
	OGX-011 plus docetaxel/prednisone	mCRPC	NCT01188187	Phase III completed
	OGX-011 plus docetaxel/prednisone	mCRPC	NCT01578655	Phase III completed
Hsp27	OGX-427 plus docetaxel	Neoplasms	NCT00487786	Phase I completed
	OGX-427	CRPC	NCT01120470	Phase II completed
	OGX-427 plus docetaxel	Relapsed or refractory metastatic bladder cancer	NCT01780545	Phase II completed
STAT3	AZD9150 plus Durvalumab	Diffuse large B-cell lymphoma	NCT02549651	Phase I completed
	AZD9150	Advanced cancers	NCT01563302	Phase I/II completed
	AZD9150	Advanced or metastatic hepatocellular carcinoma	NCT01839604	Phase I/Ib completed
	AZD9150	Malignant ascites	NCT02417753	Phase II Terminated (Could not find these types of patients)
Raf-1	LErafAON	Advanced cancer	NCT00100672	Phase I completed
	LErafAON	Advanced solid tumors	NCT00024661	Phase I completed
	LErafAON plus radiotherapy	Neoplasms	NCT00024648	Phase I completed
Raf-1/Pkc-α	ISIS 5132 plus ISIS 3521	Matastatic breast cancer	NCT00003236	Phase II completed
HIF-1α	EZN-2968	Neoplasms, liver metastases	NCT01120288	Phase I completed
	EZN-2968	Advanced solid tumors or lymphoma	NCT00466583	Phase I completed
	EZN-2968	HCC	NCT02564614	Phase I completed
	AZD4785	Advanced solid tumors	NCT03101839	Phase I completed
AR	AZD5312	Advanced solid tumors with AR pathway as a potential factor	NCT02144051	Phase I completed
c-myb	c-myb AS ODN	Hematologic malignancies	NCT00780052	Phase I completed
R2 component of mRNA	GTI-2040 plus capecitabine	mRCC	NCT00056173	Phase I/II completed
XIAP	AEG35156 plus paclitaxel	Advanced breast cancer	NCT00558545	phase I/II terminated (Avastin approved for first-in-line treatment)
	AEG35156 plus gemcitabine	Advanced pancreatic cancer	NCT00557596	Phase I/II terminated
TGF-β2	TASO-001	Solid tumor	NCT04862767	Phase I recruiting
Akt-1	WGI-0301	Advanced solid tumors	NCT05267899	Phase I recruiting
FOXP3	AZD8701 plus durvalumab	Advanced solid tumors	NCT04504669	Phase I recruiting

*Bcl-2* B-cell lymphoma 2, *Grb-2* growth factor receptor-bound protein-2, *CLU* clusterin, *Hsp27* Heat shock protein 27, *STAT3* signal transduction and transcriptional activator 3, *PKC-α* protein kinase C-alpha, *HIF-1* hypoxia-inducible factor-1, *AR* androgen receptor, *XIAP* X-linked inhibitor of apoptosis, *TGF-β2* transforming growth factor beta 2, *FOXP3* forkhead box P3, *CLL* chronic lymphocytic leukemia, *mRCC* metastatic renal cell cancer, *AML* acute myeloid leukemia, *CML* chronic myelogenous leukemia, *ALL* acute lymphoblastic leukemia, *MDS* myelodysplastic syndrome, *mCRPC* metastatic castrate resistant prostate cancer, *CRPC* castrate resistant prostate cancer, *HCC* hepatocellular carcinoma

ASOs are chemically synthesized oligonucleotides, typically 1–30 nucleotides in length, that bind to RNA following Watson–Crick base-pairing rules. The length of the ASOs allows them to bind uniquely to only one target RNA. Although the first two marketed ASO medications, Fomivirsen and Mipomersen [14], have been discontinued, there are still seven approved ASO drugs for medical use in the market [15, 16], mainly for treating diseases, such as Duchenne muscular dystrophy (DMD) [17], spinal muscular atrophy, familial amyloid polyneuropathy [18, 19], and familial chylomicronemia syndrome.

Proteins of the B-cell lymphoma 2 (Bcl-2) family play a role in the regulation of apoptosis and confer resistance to traditional cytotoxic chemotherapy and monoclonal antibodies, making Bcl-2 an attractive target for therapeutic intervention in cancers. Oblimersen sodium (Genasense™, G3139) is an antisense oligonucleotide that hybridizes to the first six codons of the open reading frame of the Bcl-2 mRNA, resulting in Bcl-2 mRNA degradation and induction of apoptosis [20]. There have been many clinical trials on oblimersen, combined with chemotherapy drugs, such as carboplatin [21, 22], paclitaxel [23], docetaxel [24–27], and irinotecan [28], for treating solid tumors. In a phase I/II trial, the combination of oblimersen and the prodrug irinotecan was well tolerated in patients with metastatic colorectal cancer; one patient experienced a partial response, and another 10 patients had stable disease lasting for 2.5–10 months (NCT00004870) [28]. Safety data from clinical trials further support the clinical development of oblimersen in combination with cytotoxic agents.

BP1001 is a liposome-incorporated antisense oligodeoxynucleotide designed to inhibit the expression of growth factor receptor-bound protein-2 (Grb-2), an essential oncoprotein in cancer cell signaling [29]. In a phase I clinical study (NCT01159028), BP1001 was well tolerated both as monotherapy and in combination with low-dose ara-C (LDAC) [30].

As a therapeutic target, clusterin is overexpressed in many cancers, inhibiting cell death pathways and modulating pro-survival and transcriptional networks [31]. OGX-011 (custirsen) is a second-generation antisense clusterin inhibitor. To determine the clinical activity of OGX-011, a randomized phase II study, in combination with docetaxel/prednisone, was conducted in patients with metastatic castration-resistant prostate cancer. Treatment with OGX-011 and docetaxel was well tolerated and associated with improved survival, as OGX-011 enhanced the tumor-killing ability of docetaxel by increasing the sensitivity of tumor cells to the drug [32]. OGX-011 may also be a new treatment strategy for patients with castration-resistant prostate cancer (CRPC) [33].

Heat shock protein 27 (Hsp27) is a stress-induced multifunctional chaperone that promotes cancer development through its proliferative and antiapoptotic functions. Hsp27 causes therapeutic resistance in prostate and other cancers, and its targeted inhibition sensitizes cancer cells to hormones and chemotherapy. OGX-427 (Apatoren) is a 2′-methoxyethyl-modified ASO that inhibits Hsp27 expression. Hsp27 participates in endoplasmic reticulum (ER) homeostasis, and the knockdown of *Hsp27* using OGX-427 induces ER stress [34]. In a phase I clinical trial, the safety profile of OGX-427 in patients with advanced cancer showed that OGX-427 was tolerated at the highest dose (1000 mg) (NCT00487786) [35].

The signal transduction and transcriptional activator 3 (STAT3) is an attractive target for many cancers. However, translating the utility of its inhibition from bench to bedside is challenging. AZD9150 (Danvatirsen, ISIS 481464), a generation 2.5 ASO, is a specific inhibitor of STAT3. Compared with generation 2.0 and previous ASOs, generation 2.5 ASOs have a higher affinity and greater intrinsic potency owing to an 8′–10′ phosphorothioate-modified deoxynucleotide “gap” flanked on either end, with 2–3 cEt nucleotides [36]. AZD9150 specifically inhibits STAT3 and induces apoptosis in various leukemia cell lines [37]. AZD9150 showed a good efficacy and safety profile in patients with heavily pretreated lymphoma and solid tumors who have undergone extensive pretreatment [38]. AZD9150 also decreased tumorigenicity and increased the chemosensitivity of neuroblastoma cells by inhibiting endogenous STAT3 and STAT3 target genes [39]. The STAT3 transcription network is an important driver of the suppressive tumor microenvironment, thus preventing checkpoint-blockade activity. In two phase I clinical studies (NCT01563302 and NCT01839604), AZD9150 monotherapy induced an immune-mediated antitumor response, suggesting that AZD9150, in combination with checkpoint-inhibitor therapy, is expected to enhance antitumor immunity [40].

LErafAON is a novel formulation of liposome-entrapped ASO targeting the Raf proto-oncogene, which encodes a factor known to play a critical role in regulating cancer cell proliferation, survival, and differentiation [41]. The preparation of LErafAON showed high liposome entrapment efficiency and stability at room temperature [42]. A phase I clinical trial evaluating its tolerability and recommended dose, in combination with radiation therapy (NCT00024648), was conducted [43]. Pharmacokinetic analysis revealed the persistence of detectable circulating rafAON at 24 h in 7 of 10 patients in the highest two-dose cohorts. Thus, liposomal formulations may promote better intratumoral AON delivery and inhibit degradation *in viv*o.

**Fig. 1:**
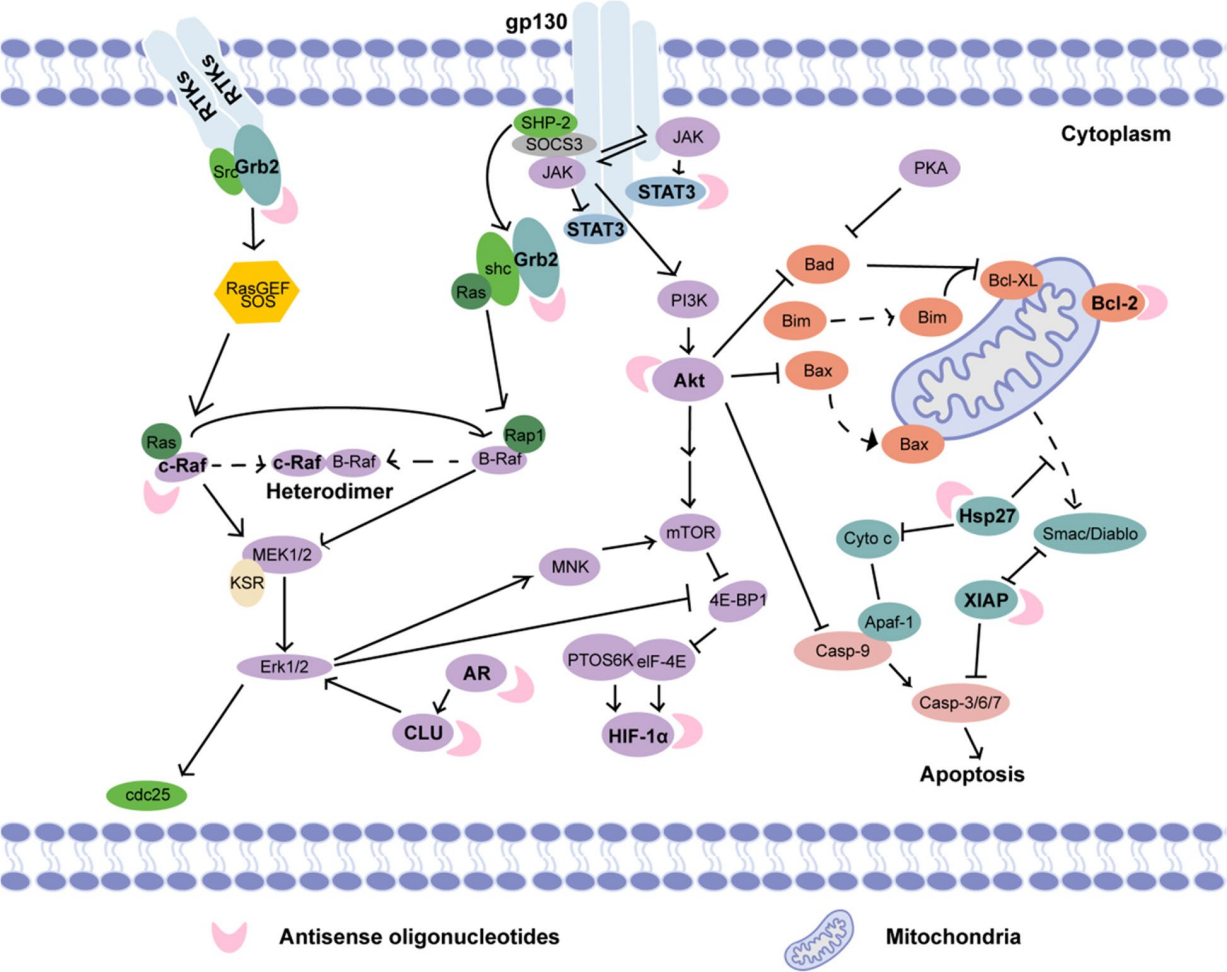
The schematic diagram of antisense oligonucleotides clinically designed for tumor targets, such as Bcl-2, Grb2, CLU, Hsp27, STAT3, c-Raf, HIF-1α, AR, and XIAP (bold font)

Hypoxia-inducible factor-1 (HIF-1) is a transcription factor that plays key roles in angiogenesis, survival, metastasis, drug resistance, and glucose metabolism. High levels of HIF-1 are associated with poor prognosis and treatment resistance in patients with solid tumors; thus, HIF-1 is an attractive target for cancer therapy. EZN-2968 (also known as RO7070179) is a third-generation ASO that specifically targets HIF-1α, a subunit of HIF-1. EZN-2968 hybridizes with HIF-1α mRNA and blocks HIF-1α protein expression in preclinical models [44]. EZN-2968 was well tolerated at the described dosage and schedule, and most toxicities reported were class 1 or 2, with no accidental toxicity [45] (Fig. 1).

### Small interfering RNA (siRNA)

Since the discovery of RNA interference (RNAi) and its subsequent application in gene knockdown in mammalian cells, siRNA therapeutics has made remarkable progress and have become promising tools against various diseases [46, 47].

As a type of noncoding double-stranded RNA (dsRNA) molecule, siRNAs are only 18–25 base pairs in length, with or without two overhanging phosphorylated bases at the 3′ end of each strand [48, 49]. As the name suggests, siRNA interferes with the expression of specific genes with complementary nucleotide sequences through mRNA degradation after transcription. Generally, siRNAs can bind to a protein complex called the RNA-induced silencing complex (RISC) in the cytoplasm. Upon binding to RISC, the guide strand is directed to the target mRNA, and the phosphodiester bond at the mRNA nucleotides 10 and 11 paired with the antisense strand is cleaved [50].

To date, four siRNA drugs (patisiran, givosiran, lumasiran, and inclisiran) have been approved for marketing to treat diseases such as TTR, acute hepatic porphyria, primary hyperoxaluria type 1, and hypercholesterolemia [51]. Patisiran [52], an siRNA drug for treating polyneuropathy in adults caused by hereditary transthyretin amyloidosis, was the first United States Food and Drug Administration (FDA)- and European Medicines Agency-approved RNAi-based therapy.siRNA drugs have potential advantages in cancer treatment compared with traditional drugs. First, as a useful therapeutic tool, siRNA can knock down genes that directly or indirectly cause abnormal proliferation of cancer cells. Thus, it is possible to treat gene-based cancers. Second, with extensive siRNA libraries available, targets for selective and specific drug development can be rapidly identified and optimized, and such target identification helps elucidate the role of specific genes in tumorigenesis. Third, the synthesis and manufacturing costs of siRNA drugs are relatively low compared to those of their antibody rivals [53]. Furthermore, optimized siRNA drugs can provide convenient dosing regimens, such as inclisiran, for biannual treatment. These advantages strongly support the notion that siRNA is among the most critical therapeutic tools for the treatment of cancers, and many siRNA drugs have been tested in clinical trials (Table 3).

**Table 3:** siRNA cancer therapeutics in clinal trials

Target	Drug name	Cancer	ClinicalTrials. gov Identifier	Current status
RRM2	CALAA-01	Solid tumor	NCT00689065	Phase I terminated
PKN3	Atu027	Advanced solid cancer	NCT00938574	Phase I completed
	Atu027	Advanced or metastatic pancreatic cancer	NCT01808638	Phase Ib/IIa completed
KRAS	siG12D LODER	Pancreatic cancer	NCT01188785	Phase I completed
	siG12D LODER	Pancreatic Cancer	NCT01676259	Phase II recruiting
	NBF-006	NSCLC, pancreatic cancer, CRC	NCT03819387	Phase I recruiting
KrasG12D mutation	KRAS G12D siRNA	Pancreatic cancer	NCT03608631	Phase I recruiting
PLK1	TKM-080301	CRC	NCT01437007	Phase I completed
	TKM-080301	HCC	NCT02191878	Phase I/II completed
EphA2	siRNA-EphA2 DOPC	Advanced or recurrent solid tumors	NCT01591356	Phase I recruiting
MYC	DCR-MYC	Solid tumors, multiple myeloma, lymphoma	NCT02110563	Phase I terminated
	DCR-MYC	HCC	NCT02314052	Phase Ib/II terminated
Bcl2L12	NU-0129	GBM	NCT03020017	Early Phase I completed
TLR9/STAT3	CpG-STAT3 siRNA CAS3/SS3	B-cell non-hodgkin lymphoma	NCT04995536	Phase I recruiting
TGF-β1/COX-2	STP705	Squamous cell carcinoma	NCT04844983	Phase II recruiting

*RRM2* M2 subunit of ribonucleotide reductase, *PKN3* protein kinase N3, *KARS* kirsten rat sarcoma, *PLK1* polo-like kinase-1, *COX-2* cyclooxygenase-2, *NSCLC* non-small cell lung cancer, *CRC* colorectal cancer, *HCC* hepatocellular carcinoma, *GBM* glioblastoma

CALAA-01, a polymer-based nanoparticle containing siRNA targeting the M2 subunit of ribonucleotide reductase (RRM2), was the first experimental RNAi-based drug screened against solid tumors by Calando Pharmaceuticals in 2008 [54]. Phase I clinical trials showed that CALAA-01 was quickly eliminated in the blood after intravenous administration and the clearance is associated with body weight [55].

Another siRNA drug, Atu027, is encapsulated inside a lipid nanoparticle (LNP) to target the protein kinase N3 (*PKN3*), an essential gene for cancer growth and metastasis [56, 57]. Clinical trial results showed that Atu027 serves a new treatment strategy for solid tumors and has good safety and activity profile in patients with advanced or metastatic pancreatic adenocarcinoma when combined with the standard chemotherapeutic gemcitabine (NCT00938574) [58].

Since the Kirsten rat sarcoma (KRAS) protein binds very closely to nucleotides, which makes it nearly impossible to identify competing nucleotide analogs, the KRAS protein has been considered undruggable for many years. Khvalevsky et al. developed a local prolonged siRNA delivery system, siG12D LODER, against mutated KRAS. This siRNA drug provides an alternative approach for controlling KRAS expression in pancreatic cancer[59]. A phase I study showed the tolerability, safety, and efficacy of siG12D LODE in patients diagnosed with pancreatic cancer and reported no obvious toxicity. Currently, siG12D LODER is undergoing phase II clinical trials [60].

TKM-080301 is an LNP formulation containing the siRNA-targeting polo-like kinase-1 (*PLK1*) gene. *PLK1* is overexpressed in hepatocellular carcinoma (HCC), and inhibition of PLK1 activity can rapidly induce mitotic arrest and apoptosis in cancer cells. TKM-080301 improved the overall survival of patients with advanced HCC [61, 62].

SiRNA-EphA2-DOPC is an siRNA drug encapsulated in neutral 1,2-dioleoyl-sn-glycero-3-phosphatidylcholine (DOPC) liposomes, which targets the ephrin type-A receptor 2 [63]. *EphA2* is overexpressed in several cancer cells. Preclinical results revealed that siRNA-EphA2 DOPC had no dose-dependent adverse effects in primates, and phase I clinical trials of siRNA-EphA2 DOPC are currently under recruitment [64].

The *MYC* oncogene family, which consists of *C-MYC*, *MYCN*, and *MYCL*, whose products regulate the transcription of at least 15% of the entire genome and participate in the growth of many solid tumor malignancies [65]. The siRNA drug DCR-MYC, designed by Dicerna Pharma, is a novel synthetic dsRNA in a stable lipid particle suspension that targets *MYC* in HCC, solid tumors, and multiple myeloma [66]. Phase I studies showed that DCR-MYC regulates tumor size in patients with solid tumors (NCT02110563).

Glioblastoma (GBM) is one of the most difficult cancers to treat because of the blood–brain and blood-tumor barriers. NU-0129, based on the spherical nucleic acid platform, is an siRNA drug designed to target the GBM oncogene Bcl2Like12 (*Bcl2L12*), which can cross the blood–brain barrier and may be a new precision medicine approach for GBM treatment. In an early phase I trial, Bcl2L12 protein levels in tumor tissues were reduced after intravenous administration of NU-0129 [67].

### MicroRNA (miRNA)

miRNA, a type of small noncoding RNAs encoded by endogenous genes approximately 19–25 nucleotides in size, participates in the regulation of post-transcriptional gene expression [68]. miRNA biogenesis occurs in the nucleus, where gene transcription is strictly regulated. Normally, once miRNAs bind to RISC to form miRISC, the relative gene expression is fine-tuned by blocking translation or cleaving the mRNA via RISC-based mechanisms, similar to those used by siRNA [69]. Thus, RSIC assembly is a key process in performing miRNA functions.

**Fig. 2:**
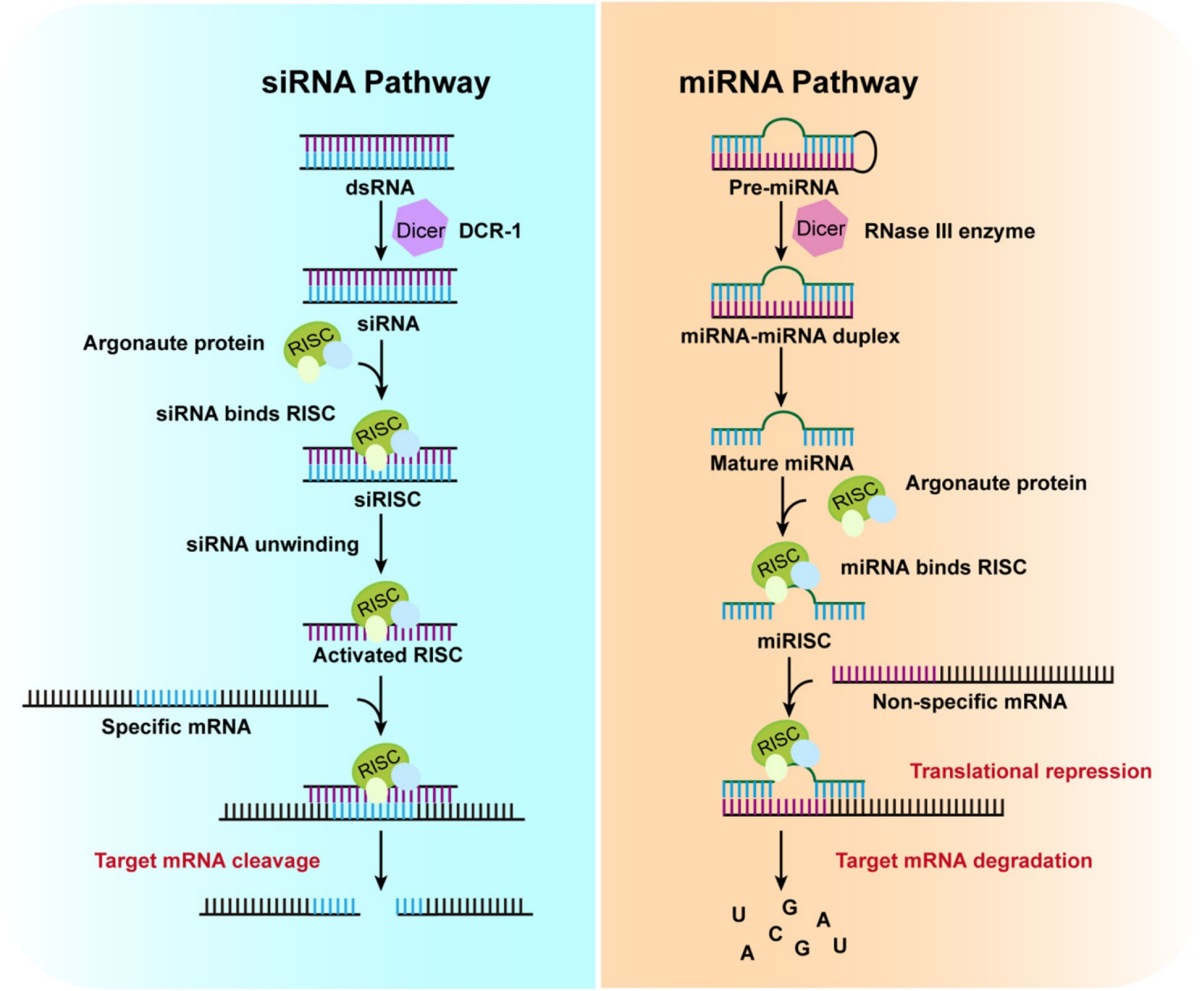
The different regulatory mechanisms of siRNA and miRNA

Although siRNA and miRNA are both noncoding RNAs with similar roles in gene silencing and regulation, siRNA is perfectly complementary to a single gene at a specific location, whereas one miRNA has multiple targets and can regulate the expression of hundreds or thousands of genes through imperfect base pairing; a gene can be regulated by several different miRNAs [70]. Thus, the clinical applications and therapeutic potential of these two are different (Fig. 2).

In 2002, miRNAs were first suggested to participate in cancer progression owing to the deletion and low expression of miR-15 and miR-16 clusters in chronic lymphocytic leukemia [71]. Over the past two decades, the association between miRNAs and various cancers has been extensively studied. miRNAs play a non-negligible role in cancer regulation, and several miRNA-based therapies are underway for different cancers. There are two strategies for miRNA-based therapeutics: miRNA mimics and miRNA inhibitors, depending on whether miRNA should be replaced or downregulated to manipulate the amount of mRNA target in the cell [72]. miRNA mimics are synthetic double-stranded oligonucleotides that can overexpress the corresponding endogenous miRNA sequence and mimic the function of the target miRNA, resulting in the downregulation of cancer cells proliferation, thereby promoting mRNA inhibition. Owing to the tumor-suppressor role of miRNAs, miRNA mimics could potentially serve as therapeutic agents for cancer management [73]. Unlike miRNA mimics, miRNA inhibitors, also known as anti-miRs, are designed as complementary single-stranded RNA analogs based on the generation of ASOs to target endogenous miRNAs. Anti-miRNAs can specifically block the upregulated expression of miRNAs associated with cancer development [74, 75].

As a potential tumor-suppressive miRNA, miR-34a is lacking in stem cells and advanced tumors. MRX34 is an LNP that can bind to miR-34a mimics[76]. MRX34 could enhance the effect of radiation therapy by inhibiting DNA repair in a non-small cell lung cancer (NSCLC) mouse model [77].

As a therapeutic target, miR-155 is a well-studied miRNA in many hematological malignancies and is mainly associated with poor prognosis in lymphoma and leukemia [78]. Cobomarsen (MRG-106), an inhibitor of miR-155, is currently undergoing clinical trials and can suppress the downstream targets or survival pathways of miR-155, including JAK/STAT, MAPK/ERK, and PI3K/AKT in vitro [79].

Remlarsen (MRG-201) was designed to mimic the activity of miR-29 and is currently being studied to determine whether it can limit the formation of fibrous scar tissues in keloids. Huang et al. found that high expression of miR-29 could regulate the STAT3 signaling pathway to inhibit the proliferation, invasion, and metastasis of uterine leiomyoma in vitro; thus, miR-29 might be a new target for treating uterine leiomyoma [80]

Several miRNA drugs have also undergone preclinical trials. For example, the miR-122 mimic could improve the sensitivity of breast cancer cells to chemotherapy drugs, such as alpelisib and trametinib, and reduce the emergence of drug resistance [81]. When the miR-151a mimic was transfected into a drug-resistant glioblastoma cell line, the cells showed miR-151a-induced enhancement of chemosensitivity to temozolomide by modulation of XRCC4-mediated DNA repair [82]. The expression level of miR-634 in gastric cancer was significantly lower than that in normal adjacent tissues, and the proliferation, migration, and invasion abilities of gastric cancer cell lines were inhibited upon transfection of the miR-634 mimic [83].

Both siRNAs and miRNAs are meaningful gene-silencing tools, and four siRNA drug candidates have been approved for marketing. However, many miRNA drugs were mostly terminated owing to safety issues, and no drug candidates have entered phase III clinical trials. Consequently, it is difficult to identify miRNAs that regulate specific genes, as they can lead to unexpected side effects. Addressing the specificity of miRNA drugs can advance the application of miRNAs in clinical settings (Table 4).

**Table 4:** miRNA cancer therapeutics in clinal trials

Target	Drug name	Cancer	ClinicalTrials. gov Identifier	Current status
miR-16	TargomiRs	MPM, NSCLC	NCT02369198	Phase I completed
miR-34a	MRX34	Primary liver cancer, solid tumors, hematologic malignancies	NCT01829971	Phase I terminated (Five immune related serious adverse events)
miR-155	Cobomarsen	Lymphoma, leukemia	NCT02580552	Phase I completed
miR-29	Remlarsen	Keloid	NCT03601052	Phase II completed

*MPM* malignant pleural mesothelioma, *NSCLC* non-small cell lung cancer

### Messenger RNA (mRNA) vaccine

mRNA, known as messenger RNA, is a single-stranded RNA complementary to the antisense DNA. It carries genetic information and directs protein synthesis in the cytoplasm [84]. As an intermediary of the central dogma of molecular biology, mRNA plays a vital role in protein production. Since Wolf et al. first successfully introduced in vitro transcription (IVT) mRNA in animals in 1900 [85], mRNA-based therapeutics, such as mRNA vaccines, have made significant progress in preventing infectious diseases and tumor immunotherapy over the past decade. In particular, because of the relatively low risk of insertion mutagenesis and lack of need to enter the nucleus for functionality, mRNA vaccines have become a hotspot in the prevention and treatment of coronavirus disease 2019 (COVID-19) caused by SARS-CoV-2 [86]. On August 23, 2021, tozinameran (Comirnaty, BNT162b2), developed by Pfizer-BioNTech, became the first mRNA vaccine officially approved for commercialization by the FDA to prevent COVID-19 among individuals aged ≥ 16 years old [87, 88]. Subsequently, the mRNA vaccine elasomeran (Spikevax, mRNA-1273), developed by Moderna, was approved for marketing [89]. These two mRNA vaccines have promoted the development of mRNA-based therapy and served as a blueprint for mRNA vaccines in cancer treatment.

Compared to other vaccines, mRNA vaccines have many advantages, such as good safety, high efficacy, shorter development cycle, and lower cost [90]. First, mRNA can be directly translated into proteins in the cytoplasm, whereas plasmid DNA and viral vectors are at risk of mutations caused by gene insertion or infection. Second, cells do not need to be involved in producing mRNA vaccines using IVT mRNA technology, thus avoiding contamination by proteins or viruses; mRNA vaccines can therefore be rapidly and economically mass-produced. In addition, based on current research data, patients showed good tolerance to mRNA vaccines, allowing repeated inoculation of mRNA vaccines.

**Fig. 3:**
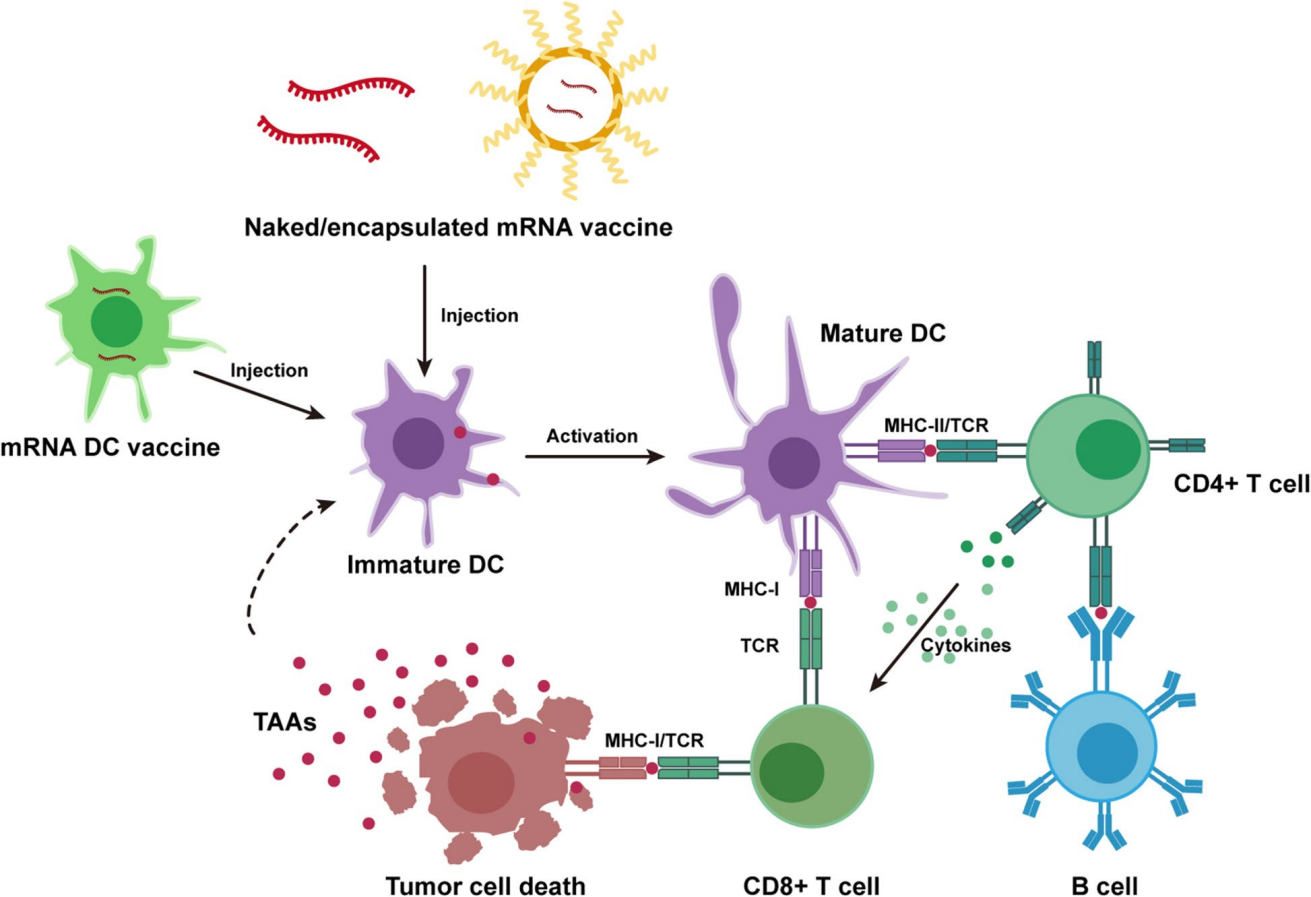
The schematic diagram of mRNA direct cancer vaccine and mRNA dendritic cell (DC) vaccine excitation of immune cells to kill tumor cell

With the development of mRNA vaccines, mRNA cancer vaccines have gradually become a research focus over the last five years (Table 5). Since cancer progression is correlated with immune response, mRNA cancer vaccines also show significant advantages in cancer immunotherapy. Through artificial design, mRNA cancer vaccines can deliver and express cancer antigens and activate innate immunity [91, 92]. Moreover, with the help of IVT mRNA technology, mRNA cancer vaccines can be used to advance personalized tumor immunotherapy. Therefore, mRNA cancer vaccines have great potential for use in antitumor therapy.mRNA cancer vaccines work by using related delivery vectors and adjuvants to deliver mRNA fragments encoding tumor antigen proteins or immunomodulatory molecules directly targeting cells. Once the tumor antigen is recognized by human immune cells, the body triggers an antitumor immune response [93]. mRNA cancer vaccines can be divided into two categories: mRNA direct cancer vaccines and mRNA dendritic cell (DC) vaccines. Using granulocyte–macrophage colony-stimulating factor (GM-CSF) as an adjuvant, mRNA direct cancer vaccines induce tumor-specific T-cell responses for tumor rejection by encoding cancer antigens, such as tumor-associated antigens (TAAs) and tumor-specific antigens. In contrast, mRNA DC vaccines obtain mRNA using IVT technology. After transfection into DCs, mRNA is translated into antigens in the cytoplasm to activate DCs, and activated DCs can present TAAs and stimulate the immune system response against tumors. Currently, there is sufficient promising preclinical evidence and many ongoing clinical trials on mRNA vaccines for cancer treatment [94] (Fig. 3).

**Table 5:** mRNA vaccine cancer therapeutics in clinal trials

Intervention	Cancer	ClinicalTrials. gov Identifier	Current status
DC vaccine	Breast cancer, malignant melanoma	NCT00978913	Phase I completed
DC vaccine	AML	NCT01734304	Phase I/II completed
DC vaccine	Melanoma	NCT00940004	Phase I/II completed
DC vaccine with mRNA from tumor stem cells	GBM	NCT00846456	Phase I/II completed
mDC vaccine/ pDC vaccine	mCRPC	NCT02692976	Phase II completed
DC vaccine plus cisplatin	Melanoma	NCT02285413	Phase II completed
DC vaccine plus docetaxel	mCRPC	NCT01446731	Phase II completed
DC vaccine	AML	NCT05000801	Recruiting
DC vaccine plus temozolomide	GBM	NCT02649582	Phase I/II recruiting
DC vaccine plus temozolomide	High grade glioma, diffuse intrinsic pontine glioma	NCT04911621	Phase I/II recruiting
DC vaccine	AML	NCT01686334	Phase II recruiting
DC vaccine plus radiotherapy and IFN-α	Malignant melanoma	NCT01973322	Phase II recruiting
RNA-loaded DC vaccine plus basiliximab	Malignant neoplasms brain	NCT00626483	Phase I completed
TriMix-DC	Melanoma	NCT01066390	Phase I completed
TriMix-DC plus ipilimumab	Melanoma	NCT01302496	Phase II completed
TriMix	Breast cancer	NCT03788083	Phase I recruiting
BTSC mRNA-loaded DCs	GBM	NCT00890032	Phase I completed
CT7, MAGE-A3, and WT1 mRNA-electroporated LCs	Multiple myeloma	NCT01995708	Phase I completed
CEA-loaded DC vaccine	CRC	NCT00228189	Phase I/II completed
MiHA-loaded PD-L-silenced DC	Hematological malignancies	NCT02528682	Phase I/II completed
mRNA transfected DC	Androgen resistant metastatic prostate cancer	NCT01278914	Phase I/II completed
GRNVAC1	AML	NCT00510133	Phase II completed
mRNA transfected DC plus docetaxel	Prostate cancer	NCT01446731	Phase II completed
Human CMV pp65-LAMP mRNA-pulsed autologous DCs	GBM	NCT02366728	Phase II completed
Human CMV pp65-LAMP mRNA-pulsed autologous DCs with or without varlilumab	GBM	NCT03688178	Phase II recruiting
pp65-shLAMP DC with GM-CSF/ pp65-flLAMP DC with GM-CSF	GBM	NCT02465268	Phase II recruiting
Autologous DCs loaded with autologous tumor RNA	Uveal melanoma	NCT01983748	Phase III recruiting
CV9103	HRPC	NCT00831467	Phase I/II completed
CV9103	HRPC	NCT00906243	Phase I/II Terminated (Study closed after completion of Phase I)
CV9104	Prostate Cancer	NCT01817738	Phase I/II terminated (Follow up period after primary analysis was prematurely stopped because more mature data will not impact the study outcome)
CV9104	Prostate Cancer	NCT02140138	Phase II terminated (Recruitment was terminated after enrolment of 35 instead of 36 evaluable patients for administrative reasons.)
CV9201	NSCLC	NCT00923312	Phase I/II completed
CV9202 and local radiation	NSCLC	NCT01915524	Phase I terminated (Slow recruitment in stratum 3: enrolled only 2 instead of 8 pts. within predicted time)
CV9202 plus durvalumab and tremelimumab	NSCLC	NCT03164772	Phase I/II completed
mRNA-5671/V941 with or without pembrolizumab	KRAS mutant advanced or metastatic NSCLC, CRC or pancreatic adenocarcinoma	NCT03948763	Phase I completed
mRNA-2416 plus durvalumab	Advanced malignancies	NCT03323398	Phase I/II terminated (This study was halted prematurely because the efficacy endpoints were not met for either treatment arm.)
mRNA-4157 plus Pembrolizumab	Melanoma	NCT03897881	Phase II active, not recruiting
mRNA-4157 plus pembrolizumab	Solid tumors	NCT03313778	Phase I recruiting
mRNA-2752 plus durvalumab	Advanced or metastatic solid tumor malignancies or lymphoma	NCT03739931	Phase I recruiting
mRNA-4359 plus pembrolizumab	Advanced solid tumors	NCT05533697	Phase I/II recruiting
mRNA RNA loaded lipid particles	GBM	NCT04573140	Phase I recruiting
OTX-2002	HCC and other solid tumor types known for association with the *MYC* oncogene	NCT05497453	Phase I/II recruiting
BNT141 plus nab-paclitaxel and gemcitabine	Advanced unresectable or metastatic CLDN18.2-positive solid tumors	NCT04683939	Phase I/II recruiting
BNT113	HPV16 + head and neck cancer	NCT03418480	Phase I/II recruiting
BNT113 plus pembrolizumab	Unresectable head and neck SCC	NCT04534205	Phase II recruiting
BNT111	Melanoma	NCT02410733	Phase I active, not recruiting
BNT111 plus cemiplimab	Unresectable Stage III or IV melanoma	NCT04526899	Phase II recruiting
Stabilized tumor-mRNA plus GM-CSF	Malignant melanoma	NCT00204607	Phase I/II completed
mRNA coding for melanoma associated antigens plus GM-CSF	Malignant melanoma	NCT00204516	Phase I/II completed
Personalized cellular vaccine	Brain cancer	NCT02808416	Phase I completed
Neoantigen tumor vaccine with or without PD-1/L1	Advanced gastric cancer, esophageal cancer, and liver cancer	NCT05192460	Recruiting
Neoantigen mRNA personalised cancer SW1115C3	Advanced malignant solid tumors	NCT05198752	Phase I recruiting
RO7198457 with or without atezolizumab	Advanced or metastatic tumors	NCT03289962	Phase I active, not recruiting
RO7198457 plus atezolizumab and mFOLFIRINOX	Pancreatic cancer	NCT04161755	Phase I active, not recruiting
RO7198457 plus pembrolizumab	Advanced melanoma	NCT03815058	Phase II active, not recruiting
RO7198457	Stage II and stage III colorectal cancer	NCT04486378	Phase II recruiting
HB-201	HPV 16 + confirmed oropharynx cancer, cervical cancer	NCT04630353	Early Phase 1 recruiting
GRANITE (GRT-C901/GRT-R902)	Colon cancer	NCT05456165	Phase II recruiting

*DC* dendritic cell, *mDC* myeloid dendritic cells, *pDC* plasmacytoid dendritic cells, *LCs* langerhans cells, *GM-CSF* granulocyte–macrophage colony-stimulating factor, *CEA* carcinoembryonic antigen, *mCRPC* metastatic castration-resistant prostate cancer, *AML* acute myeloid leukemia, *GBM* glioblastoma, *mCRPC* metastatic castration-resistant prostate cancer, *CRC* Colorectal cancer, *AML* acute myelogenous leukemia, *HRPC* hormonal refractory prostate cancer, *NSCLC* non-small cell lung cancer, *HCC* hepatocellular carcinoma, *CLDN18.2* Claudin 18.2, *SCC* squamous cell carcinoma

TriMixDC is an autologous monocyte-derived DC electroporated with mRNA encoding a mixture of three immune-modulating molecules, including active TLR-4, CD40 ligand, and CD70 [95], which can stimulate T cells. TriMixDC-MEL, obtained by co-electroporation of TriMixDC with an mRNA encoding melanoma-associated antigens, showed favorable safety, strong immunogenicity. It produced a durable tumor response in 4 of 15 patients with advanced melanoma after intravenous and intradermal combined administration (NCT01066390) [96]. When combined with the immune-checkpoint blocker ipilimumab to overcome immune tolerance, the median progression-free survival and overall survival rates improved in patients with advanced melanoma treated with TriMixDC-MEL, and robust CD8^+^ T-cell responses were detected (NCT01302496) [97].

CV9103 and CV9104 are both mRNA-based vaccines based on RNActive® technology. CV9103 encodes four specific antigens present in cancer cells: prostate-specific antigen (PSA), prostate-specific membrane antigen (PSMA), prostate stem cell antigen (PSCA), and six-transmembrane epithelial antigen of the prostate (STEAP). The follow-up vaccine CV9104 encodes six antigens, i.e., PSA, PSMA, PSCA, STEAP, mucin 1, and prostatic acid phosphatase (PAP), which are overexpressed in prostate cancer cells compared to those in healthy tissues [98]. These antigens are appropriate targets for intervention and can induce adaptive immunity in humans. In a phase I/II clinical trial, CV9103 displayed safety and activated immunogenicity in patients with advanced prostate cancer, and one patient showed a confirmed PSA response [99]. However, the subsequent trial with CV9104 for prostate cancer was terminated because there was no significant improvement in overall survival compared to that in patients treated with placebo [100].

CV9201 is another mRNA vaccine based on RNActive®, which encodes five NSCLC antigens, including melanoma antigen family C1 (MAGE-C1), MAGE family C2, New York esophageal squamous cell carcinoma 1 (NY-ESO-1), trophoblast glycoprotein (5T4), and survivin. CV9201 showed an acceptable tolerability profile and evidence of immune activation in a phase I/IIa dose-escalation experiment (NCT00923312) [101]. CV9202 is also a self-adjuvanted mRNA vaccine encoding six NSCLC-associated antigens, namely NY-ESO-1, MAGE-C1, MAGE-C2, survivin, 5T4, and MUC-1, which induce targeted immune responses. A phase Ib clinical trial demonstrated that treatment with CV9202 combined with radiotherapy in 26 patients with stage IV NSCLC was well tolerated, and antigen-specific immune responses were detected in 84% of patients (NCT01915524) [102]. Further clinical trials on CV9202 evaluating its safety and preliminary efficacy, combined with the immune checkpoint inhibitors durvalumab (anti-PD-L1) or remelimumab (anti-CTLA-4), have been conducted (NCT03164772) [103].

BNT111 is an intravenously administered tetravalent liposomal RNA vaccine encoding four TAAs: NY-ESO-1, melanoma-associated antigen A3 (MAGE-A3), tyrosinase, and transmembrane phosphatase with tensin homology. These antigens show restricted normal tissue expression, high immunogenicity, and high prevalence in melanoma. When entering the body, BNT111 is taken up by antigen-presenting cells (APCs), translocated to the cytoplasm, and translated into four tumor-associated proteins, ultimately triggering antigen-specific CD8^+^ and CD4^+^ T cell responses. A first-in-human dose-escalation phase I clinical study showed that BNT111 exhibited good safety and induced durable objective immune responses in patients with advanced melanoma (NCT02410733) [104]. An open-label, randomized, multicenter phase II trial is currently ongoing to evaluate the safety, tolerability, and efficacy of BNT111, in combination with cemiplimab, in patients with unresectable stage III or IV melanoma with anti-PD-1-refractory or relapse after anti-PD-1 therapy (NCT04526899).

Autogene cevumeran, also called RO7198457, consists of RNA-Lipoplex (RNA-LPX) and is an individualized neoantigen-specific therapy (iNeST) that can potentially stimulate and expand neoantigen-specific CD4^+^ and CD8^+^ T cells, leading to antitumor responses. Currently, four clinical trials are underway or under recruitment. One is a first-human phase I study designed to evaluate the safety, tolerability, immune response, and pharmacokinetics of RO7198457 as a single agent or in combination with the anti-PD-L1 antibody atezolizumab in participants with locally advanced or metastatic tumors (NCT03289962). A randomized phase II study of RO7198457 in combination with pembrolizumab was conducted in patients with previously untreated advanced melanoma (NCT03815058).

### Challenges

Despite considerable progress in RNA-based therapeutics, two major challenges remain for clinical application: selecting the best drug target from a large number of possible targets and optimizing the delivery of RNA drugs to individual tumors [105]. The choice of targets and delivery routes can enhance drug efficacy while minimizing side effects in normal tissues and increasing drug safety.

### Target

Cancer is caused by a variety of complex factors, including genetic lesions. Many small-molecule therapeutics directly target key genetic genes for cancer treatment. In RNA-based drug development, we should seriously consider potential genetic targets and concentrate on those that are difficult to target using small molecules. For example, the *MYC* oncogene family is frequently deregulated in most human cancers and is associated with poor prognosis and unfavorable patient survival [65]. One of the potential ways to treat cancer is to inhibit *MYC* expression; however, owing to the disorderly structure of the MYC protein, there is currently no small-molecule inhibitor with good activity and high selectivity that directly targets *MYC *[106].

*KRAS* is among the most common oncogenes in solid tumors. However, few *KRAS*-targeted drugs are currently available. Currently, only Lumakras (Sotorasib, Amgen), approved by the FDA on May 28, 2021, is used to treat patients with a proto-oncogene *KRAS* G12C-mutated NSCLC, the first targeted drug approved for *KRAS* mutations [107]. Therefore, these oncogenes can be preferred targets against which oligonucleotide drugs can be developed.

Cancer is a multifactorial disease that involves multiple genes. Thus, targeting only one associated gene may be insufficient. Combination therapies that simultaneously target multiple affected genes can be a viable approach in the future. Oligonucleotide therapeutics are particularly amenable to combination therapy because the same drug modality can be applied to target multiple cancer drivers [12].

Although neoantigens have shown great potential in cancer immunotherapies, identifying suitable cancer neoantigens that can be targeted by mRNA vaccines remains a challenge. Alternative splicing occurs widely in tumors and has been proven to contribute to the generation of candidate neoantigens [108]. However, abnormal alternative splicing occurs in many tumors, which may lead to the translation of abnormal transcripts into tumor-specific proteins. High-throughput technologies enable systematic characterization of alternative splicing and may identify alternative splicing-derived cancer neoantigens from RNA-seq data. It is also possible to design personalized mRNA vaccines based on alternative splicing-derived cancer neoantigens [109].

### Delivery

Currently, delivery is among the greatest barriers to the widespread application of RNA-based therapeutics. In particular, safe, efficient, and targeted delivery of oligonucleotide drugs and mRNA vaccines remains a major challenge [16, 110] (Table 6). First, naked and unmodified RNAs are poorly stable, easily degraded by multiple circulating ribonucleases (RNases) and hydrolases, and rapidly cleared by renal clearance upon systemic injection. Second, as a hydrophilic negatively charged macromolecule, oligonucleotide drugs have limited ability to penetrate cell membranes, making it difficult to enter the cytoplasm or nucleus. In addition, ASO and siRNA sequences may have off-target effects, leading to non-specific gene knockdown and activation of the innate immune system via Toll-like receptors. Thus, optimized RNA drug delivery systems can protect RNA structures from degradation, increase targeting capacity, and reduce toxic side effects.

**Table 6:** Currently developed delivery platforms in RNA therapeutics

Delivery platform	Classification	Pros	Cons
Viral vectors	Adenovirus, adeno-associated virus, lentivirus	High transfection efficiency	Immunogenicity, high cost, toxicity
Lipid-based delivery system	Micelles, liposomes, lipid nanoparticles	Easy to production, lack of immunogenicity, biodegradability	Difficult to large-scale
Polymer-based nanoparticles	Cationic polymers, dendrimers	Small size, low immunogenicity and toxicity	Poor biodegradability
Inorganic nanoparticles	Gold nanoparticles, silica nanoparticles, carbon nanotubes	Easy functionalization, good biocompatibility, high load capacity, mass production	Limited transfection efficiency, lack of clinical trials

With the development of feasible technologies that improve the druggability of RNA molecules, various viral and non-viral delivery systems have emerged. Currently, there are three key viral vectors for gene therapy: adenovirus (AdV), adeno-associated virus (AAV), and lentivirus [111]. Over the past two decades, they have achieved preclinical and clinical successes. AAV was first identified in laboratory AdV preparations in the mid-1960s [112]. Recombinant AAV is also a leading platform for in vivo delivery of gene therapies [113].

However, viral vectors pose toxicity issues and are unsafe for humans owing to their inflammatory and immunogenic effects, which limit their clinical translation [114]. Compared with viral vectors, non-viral vectors have a wider range of application, and they have overcome some issues, including high cost, immunogenicity, and toxictity [115]. Therefore, relatively safe non-viral vectors, such as lipid-based delivery systems, polymer-based nanoparticles, and inorganic nanoparticles, are rapidly evolving [116].

Lipid-based delivery systems, such as micelles, liposomes, and LNP, can be easily synthesized through chemical reactions [117, 118]. The efficiency of delivering RNA therapy to the liver is greatly improved by distinct chemical structures and more reasonable lipid molecular design. LNPs are one of the most widely used non-viral delivery systems for oligonucleotide drugs and mRNA vaccines, and their advantages include ease of production, biodegradability, protection of the embedded RNA from RNase degradation and renal clearance, promotion of cellular uptake, and endosomal escape [119, 120]. Recently, LNP has received global attention as an important component of mRNA vaccines, playing a key role in effectively protecting and transporting mRNA into cells. Polymers are the second largest class of nucleic acid-delivery vehicles after lipids. Cationic polymers form stable complexes with anionic nucleic acids, providing a versatile, scalable, and easily adaptable platform for efficient nucleic acid delivery while minimizing the immune response and cytotoxicity [121]. The efficiency of RNA delivery into cells can be altered by adjusting polymer polarity, degradation, and molecular weight. Dendrimers are another type of polymer that deliver RNA [122]. These macromolecules are centered on a core molecule and synthesize highly branched polymers via repetitive growth reactions. Modifying the dendrimer structure can protect nucleotides from enzymatic degradation.

With the development of nanomaterials, inorganic nanocarriers provide a unique platform for the effective delivery of nucleic acid drugs to tumor cells due to their high stability, good biocompatibility, low immunogenicity, and mass production, such as gold nanoparticles (AuNPs) [123, 124], silica nanoparticles [125], and carbon nanotubes. AuNPs are [126] a classical inorganic nanocarrier with good chemical stability and biocompatibility [127]. Nucleic acid chains are covalently attached to the AuNP core via mercaptan groups. The abovementioned NU-0129 is a siRNA drug designed based on AuNPs to target the oncogene *Bcl2L12* in GBM treatment. Silica is another type of biodegradable, safe, and stable carrier nanomaterial. Mesoporous silica nanoparticles (MSNs) have attracted great interest for their easy functionalization, biocompatibility, high specific surface area, and biodegradability [128]. MSNs can effectively deliver drugs to cells and easily escape from endosomes, thereby enhancing anti-tumor effects [129]. Bertucci et al. co-delivered anti-miR-221 PNA and temozolomide to induce drug-resistant glioma cell apoptosis by using MSNs [130].

Viral vectors are more effective but more immunogenic than non-viral delivery systems. Non-viral gene vectors are generally versatile, simple, cost-effective, and potentially safer alternatives but may lack adequate clinical efficacy. Therefore, when selecting a delivery vehicle for an RNA drug, it is necessary to consider many aspects and select the most suitable one to maximize efficacy and minimize side effects.

## Conclusion

RNAs can be used both as a target and a drug. The successful development of various new oligonucleotide drugs and mRNA COVID-19 vaccines has resulted in an increasing number of RNA-based drugs that show great promise for clinical translation. RNA therapy offers an innovative approach to new drugs for cancer treatment, with several important advantages, including high specificity for the target, modular development by replacing RNA sequences, predictability in terms of pharmacokinetics and pharmacodynamics, and relative safety. However, some challenges are associated with this therapy, including the selection of suitable targets, innovation, and optimization of delivery systems.

Although these non-protein-targeted drugs have certain limitations, the market potential of RNA therapeutics in the treatment of tumors and other diseases cannot be ignored, along with the continuous breakthrough of core technologies, such as chemical modification and delivery systems. The successful commercialization of oligonucleotide drugs and mRNA vaccines has promoted a wave of nucleic acid drug research and development, and large-scale production and economic benefits have now become the main focus point. Non-protein-targeted drugs can overcome the limitations of the druggability of small molecule and antibody drugs and are thus expected to become the third major drug type.

With a deeper understanding of the multiple types and functions of RNA, the ability to generate modified RNAs with higher stability and drug activity, and nanotechnology-based vectors capable of targeted delivery of these RNAs into cells, the development of targeted RNA therapeutic options with multiple specificities is expected to change the landscape of cancer treatment in humans.
